# Strontium ranelate causes osteophytes overgrowth in a model of early phase osteoarthritis

**DOI:** 10.1186/s12891-017-1399-2

**Published:** 2017-02-10

**Authors:** Jian-Guo Chu, Mu-Wei Dai, Yu Wang, Fa-Ming Tian, Hui-Ping Song, Ya-Ping Xiao, Li-Tao Shao, Ying-Ze Zhang, Liu Zhang

**Affiliations:** 1 Department of Orthopedic Surgery, Hebei Medical University, Shijiazhuang, China; 2 Medical Research Center, North China University of Science and Technology, Tangshan, China; 3 Department of Orthopedic Surgery, The Affiliated Hospital of North China University of Science and Technology, Tangshan, China

**Keywords:** Osteoarthritis, Strontium ranelate, Cartilage, Osteophytes, Subchondral bone, Micro-CT

## Abstract

**Background:**

Osteoarthritis (OA) involves cartilage changes as well as modifications of subchondral bone and synovial tissues. Strontium ranelate (SR), an anti-osteoporosis compound, which is currently in phase III clinical trial for treatment of OA. Evidences suggest that SR preferably deposited in osteophyte, other than in subchondral bone in early phase of OA. This phenomenon raises concern about its utility for OA treatment as a disease-modifying drug. To evaluate the effect of SR on cartilage, subchondral bone mass and subchondral trabecular bone structure in medial meniscectomized (MNX) guinea pigs.

**Method:**

Thirty-six 3-month-old male Dunkin Hartley albino guinea pigs received either sham or medial meniscectomy operations. One week after the procedure, meniscectomized animals began 12 weeks of SR (625 mg/kg, daily) treatment by oral gavage for MNX + SR group, or normal saline for MNX + V group. All animals were euthanized 12 weeks later, cartilage degeneration and subchondral bone micro-architecture was analyzed.

**Results:**

Both OARSI scores (*P* = 0.523 for marcoscopic scores, *P* = 0.297 for histological scores) and Cartilage thickness (*P* = 0.335) in MNX + SR group were comparable to MNX + V group. However, osteophyte sizes were larger in MNX + SR group (*P* = 0.014), and collapsed osteophytes in MNX + SR group (7 by 12) were significantly more than in MNX + V group (1 by 12) (*P* = 0.027), while immunohistochemistry indicates catabolic changes in osteophyte/plateau junction. Micro-CT analysis showed bone mineral density (BMD) (*P* = 0.001), bone volume fraction (BV/TV) (*P* = 0.008), trabecular spacing (Tb.Sp) (*P* = 0.020), trabecular thickness (Tb.Th) (*P* = 0.012) and structure model index (SMI) (*P* = 0.005) levels to be significantly higher in the MNX + SR group than in the MNX + V group.

**Conclusions:**

SR (625 mg/kg/day) did not protect cartilage from degeneration in MNX guinea pigs but subchondral bone was significantly enhanced. In early phase OA, SR administration causes osteophyte overgrowth, which may be related to incorporation into mineralizing osteophytes. This adverse effect is important for future studies of SR in OA.

## Background

Osteoarthritis (OA) involves cartilage changes as well as modifications of subchondral bone and synovial tissues. Evidence suggest that subchondral bone mass is reduced in early progression of OA [1, 2]. In contrast, some anti-resorptive drugs, such as bisphosphonates [3] and PTH(1-34) [4], were found to protect cartilage from OA progression, so various bone metabolism regulators were considered for potential disease-modifying OA drugs and many are under evaluation [5–7].

Strontium ranelate (SR) an anti-osteoporosis compound that protects postmenopausal women from vertebral and non-vertebral fractures [8], it is currently in phase III clinical trial for treatment of OA [9]. It contains strontium element (Sr) which is similar to calcium in that it can be incorporated into bone and/or stimulate calcium-sensing receptors in bone cells [10]. In vitro data show that SR stimulates OPG and inhibits RANKL synthesis in osteoblasts [11]. Several clinical studies indicated that OA patients would benefit from SR treatment. SR has been shown to reduce a urinary cartilage degradation biomarker, CTX-II, in postmenopausal women [12]. Another double-blind, randomized, placebo-controlled trial confirmed that SR 2 g/day is associated improvements in Western Ontario and McMaster Universities Osteoarthritis Index (WOMAC) scores as evidenced by radiographic data [13]. Another trial indicates treatment with strontium ranelate 2 g/day over 3 years is associated with a clinically meaningful improvement in pain from 6 months after treatment initiated [14]. Animal models are also underway to investigate the mechanism of SR in OA, which have shown therapeutic effects [15–17].

However, several recent studies [6, 18] showed evidences that SR preferably deposited in osteophyte, other than in subchondral bone. This phenomenon raises concern about its utility for OA treatment as a disease-modifying drug, which also prompts that it still needs to study on the effect of SR on OA joints. Thus we studied the effect of SR in a meniscectomized (MNX) guinea pig model, however, found a potential adverse effect of SR in this early phase OA model.

## Methods

### Animal handling

All experiments were approved by the University’s Animal Care and Use Committee. Thirty-six 3 month-old male specific-pathogen free grade Dunkin Hartley (DH) albino guinea pigs (Vital River Experimental Animal Technical Co., Ltd., China) were housed in pairs and given one week to acclimate to the housing facility. At the start of the experiments, animals weighed 736 ± 65 g (mean ± SD). Environmental conditions were 25 °C ± 1 °C, humidity 55% ± 10%, with a 12:12 light: dark cycle with lights on at 07:00 and off at 19:00. Animals were housed in 545 × 380 × 200 mm cages and given access to a sterilized diet (^60^Co Guinea Pig Diet 3035, Beijing HFK Bioscience Co., Ltd., China) and water ad libitum. Environmental enrichment was four handfuls of sterilized sawdust nesting material. During housing, animals were monitored once daily for health and environmental maintenance. No adverse events were observed.

Animals were randomly divided into three groups as follows: Sham group, MNX + V, and MNX + SR groups, twelve animals per group. Each group was then randomly divided into two subgroups for macroscopic scoring/micro CT testing (*n* = 6) or histological scoring/immunohistochemistry assays (*n* = 6). Animals were anesthetized with sodium pentobarbital (30 mg/kg, ip), and a medial meniscectomy was performed on the right knee to create MNX + V, and MNX + SR groups according to a protocol described by Bendele [19]. A sham operation consisting of only an incision in the skin at the same location was performed on sham animals. Animals were all carefully handled and kept warm during and after surgery. Drug administration was initiated one week after surgery. The animals in MNX + SR group received Osseor (Servier, Co., France) by oral gavage at a dosage of 625 mg/kg daily at 9:00 AM. Normal saline was given to MNX + V group as a placebo control and treatment lasted for 12 weeks. Body weights did not differ among groups by the end of the experiment. All animals were euthanized by intraperitoneal overdose injection of sodium pentobarbital, blood and operated knee joints were harvested. All reasonable efforts were made to minimize pain and suffering. No surgery failed and no animals died during the experiment.

### Specimen processing and OARSI scoring

Primary outcomes were defined using Osteoarthritis Research Society International (OARSI) scores, and other results were designated as secondary outcomes. All samples for macroscopic scoring were sequentially handled according to our previous research [20]. The gross appearances were documented by a digital camera (Canon 550D, Canon, Japan) for evaluating the stage of the disease according to OARSI macroscopic scoring system in a blinded fashion [21]. Specimens were then processed for micro-Computed Tomographic (micro-CT) assessment.

All samples used for histological assessment were also handled according to our previous research to produce 8 μm coronal knee sections [20]. Three non-consecutive toluidine blue stained sections from each sample were analyzed under an optical microscope (40×, Olympus BX53, Olympus, Japan) for semi-quantitative OARSI microscopic scoring [21].

**Fig. 1:**
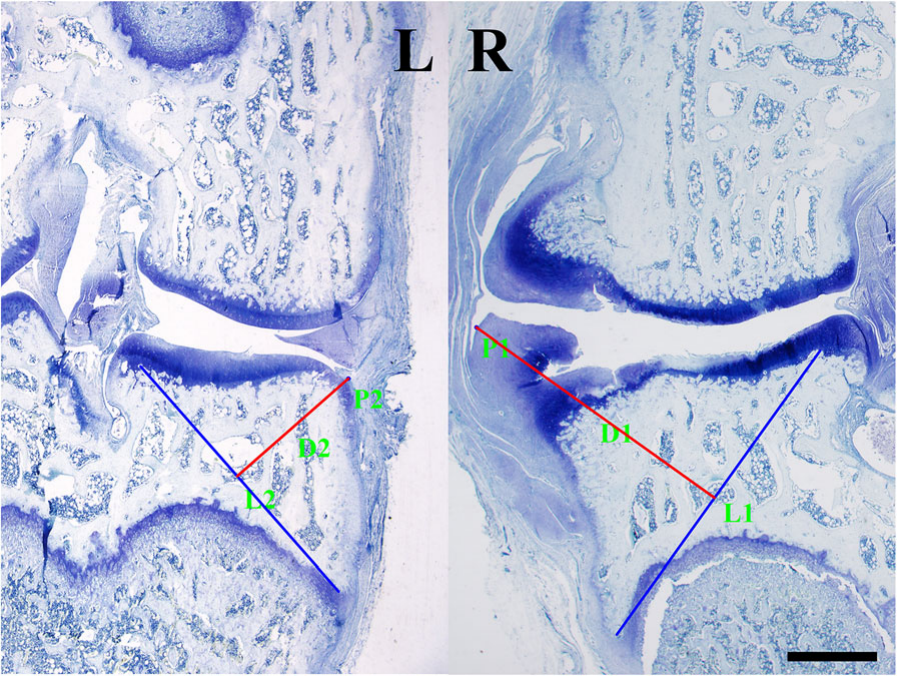
Methods for measuring osteophytes on medial tibial plateau of both sides of knee. Notes: The *blue line* (L1 or L2) is the line reference drawn from the medial intercondylar eminence to the lower corner of the epiphyseal cancellous bone. The distance between the outer upper edge of the osteophyte (P1 or P2) and the reference line was indicated by the *red line* (D1 or D2). The *bar* represents length of 1mm

Considering osteophytes formed on the edge of guinea pig joints consist both cartilaginous tissue and bony tissue, osteophytes were measured as indicted in Fig. 1. A line reference was drawn from the medial intercondylar eminence to the lower corner of the epiphyseal cancellous bone for each sample (L1 or L2). Then, the distance between the outer upper edge of the osteophyte (P1 or P2) and the reference line for the operated knee (D1) and the non-operated knee (D2) was measured. Finally, osteophytes represented by differences in the distances between both knees from the same animal (D1-D2) were noted. Additionally, a collapsed osteophyte could be seen in Fig. 1, which was defined by disruption of continuity and hollow space in cartilaginous tissue on the edge of tibial plateau. Scoring and measurements were performed in a blinded fashion and averages of the three sections were representative data.

### Subchondral bone microstructure measurement

Proximal tibiae were scanned using a micro-CT system (ZKKS-Sharp-MCT, Guangzhou, China) to quantify the micro-architecture of the subchondral trabecular bone, and the volume of interest (VOI) was defined as the epiphyseal cancellous bone region 0.5 mm beneath the subchondral plate of medial tibiae, with voxel size of 20 μm. The peak X-ray tube potential was 40 kVp and its intensity was 250 mA, respectively. Bone mass and structural characteristics of VOI were described using bone mineral density (BMD), bone volume fraction (BV/TV), trabecular number (Tb.N), trabecular spacing (Tb.Sp), trabecular thickness (Tb.Th), structure model index (SMI), and degree of anisotropy (DA). All data were calculated using software designed for the machine.

### Immunohistochemical assessment

For further investigation of cartilage status, aggrecan (AGG), collagen-II (Col-II), caspase-3, a disintegrin and metalloproteinase with thrombospondin motifs 4 (ADAMTS-4), and metalloproteinase-13 (MMP-13) were detected using immunohistochemistry. Paraffin sections were deparaffinized, rehydrated, and subjected to routine antigen retrieval using 0.05% trypsin at 37 °C for 30 min, and endogenous peroxidase activity was suppressed by 0.3% H_2_O_2_ for 15 min, then incubated overnight at 4 °C with the following antibodies: aggrecan (1:200) (PAB908Ra02, Cloud-Clone Corp., U.S.), ADAMTS-4 (1:200) (ab185722, Abcam Inc., U.S.), Caspase-3 (1:200) (PAA626Ra01, Cloud-Clone Corp., U.S.), Col-II (1:50) (II-II6B3 was deposited to the DSHB by Linsenmayer, T.F.), and MMP-13 (1:200) (PAA099Ra01, Cloud-Clone Corp., U.S.), respectively. Other procedures were performed according to instructions provided with the respective PV-6001 Polink-1 HRP DAB Detection System (ZSGB-BIO Corp., China) and ZLI-9017 DAB kit (ZSGB-BIO Corp., China). Sections were counterstained using Harris’ hematoxylin solution (BASO Diagnostics Inc., China) for 30 sec and target protein measurements in cartilage tissue of the tibial plateau was evaluated by the average intensity of optical density. The average intensity of optical density, given in IOD/mm^2^, was defined as the sum of integrated option density divided by area of cartilage tissue in the ROI under a magnification of 100×. The ROI was defined as the medial tibial plateau cartilage that was 0.5 mm from the medial intercondylar eminence. These procedures were performed using Image-Pro Plus (Media Cybernetics, Inc., U.S.).

### Statistical analysis

All data are expressed as means with 95% confidence intervals and categorical data were analyzed using a Chi-square test with the Fisher’s exact test. A Kolmogorov-Smirnov test was used to confirm whether distribution of numerical data fulfilled Gaussian distribution. Comparisons of Gaussian distributed measurements between groups were tested using ANOVA. Fisher’s least significant difference *t*-test or Dunnett’s T3 test was used to compare any two groups, based on homogeneity of variance. A Kruskal-Wallis H test was used to analyze OARSI scores and non-Gaussian distributed data, then a Mann-Whitney *U* test was used to compare all pairs of groups. Two-tailed values of *P* < 0.05 were considered statistically significant. All statistical analyses were processed using SPSS software (SPSS 17.0, SPSS Inc.; Chicago, IL, U.S.).

## Results

### OARSI scoring of cartilage

**Fig. 2:**
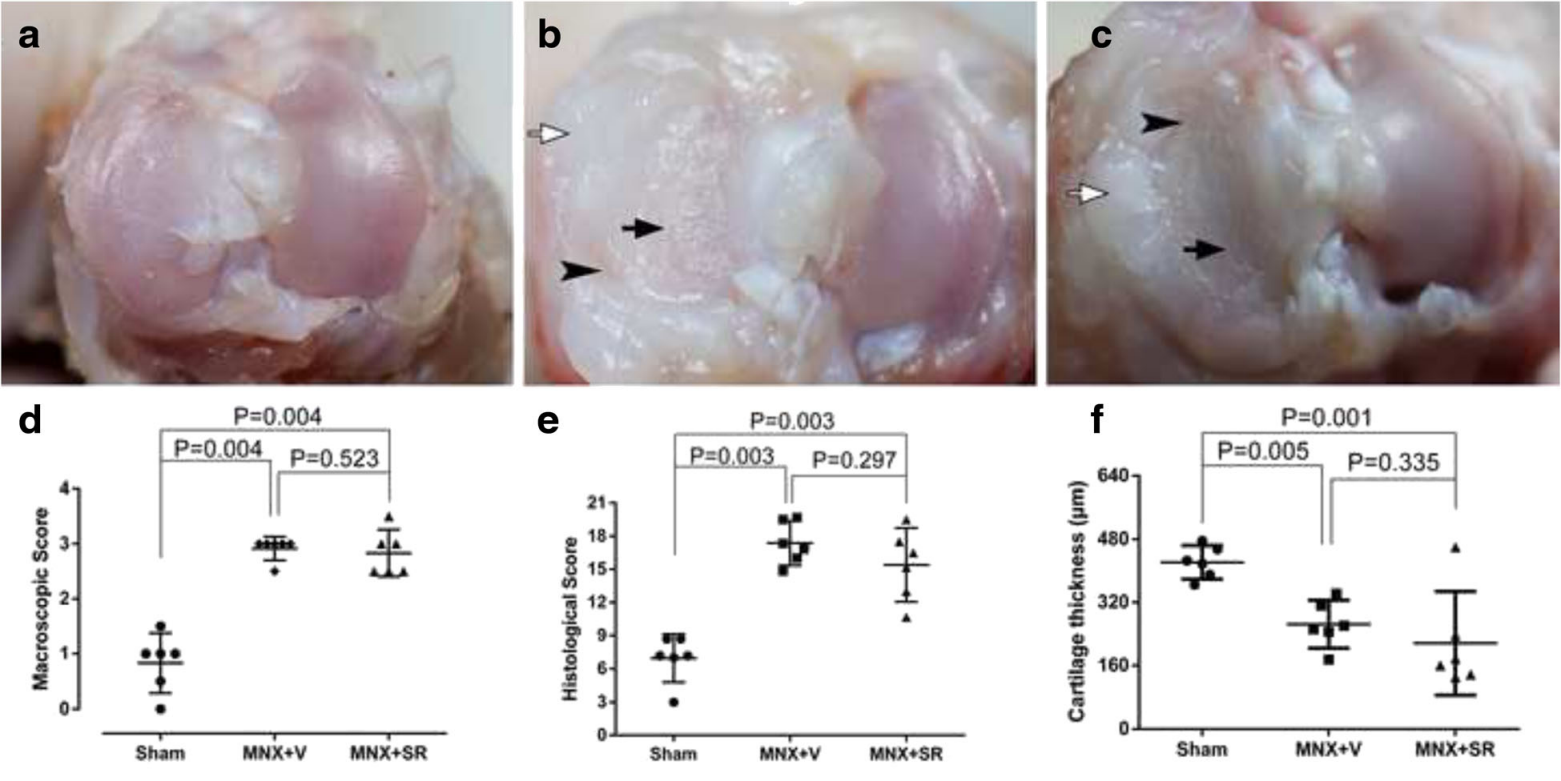
Macroscopic view of right tibial plateau of samples and OARSI macroscopic scoring between groups. Notes: Sham group: (**a**); MNX + V group: (**b**); MNX + SR group: (**c**); OARSI macroscopic scores between groups (**d**); OARSI histological scores between groups (**e**); The results of cartilage thickness (**f**). *Black arrow* indicate cartilage leisions, *black arrow* head indicate clefts on cartilage, *white arrows* indicate chondral hyperplasia

The induction of OA significantly impaired articular cartilage of the medial tibial plateau which become rougher and thinner than that in the sham group, with clefts and chondral hyperplasia on the medial edge of the cartilage (Fig. 2). Five MNX + SR samples had collapsed osteophytes, and one sample was similar to this in the MNX + V group, but there were no significant differences between the MNX + SR and MNX + V groups (*P* = 0.080). These results and OARSI macroscopic scores indicated that joints operated on had higher scores than sham-operated joints but there were no significant differences in macroscopic scores between MNX + SR and MNX + V groups (*P* = 0.523).

**Fig. 3:**
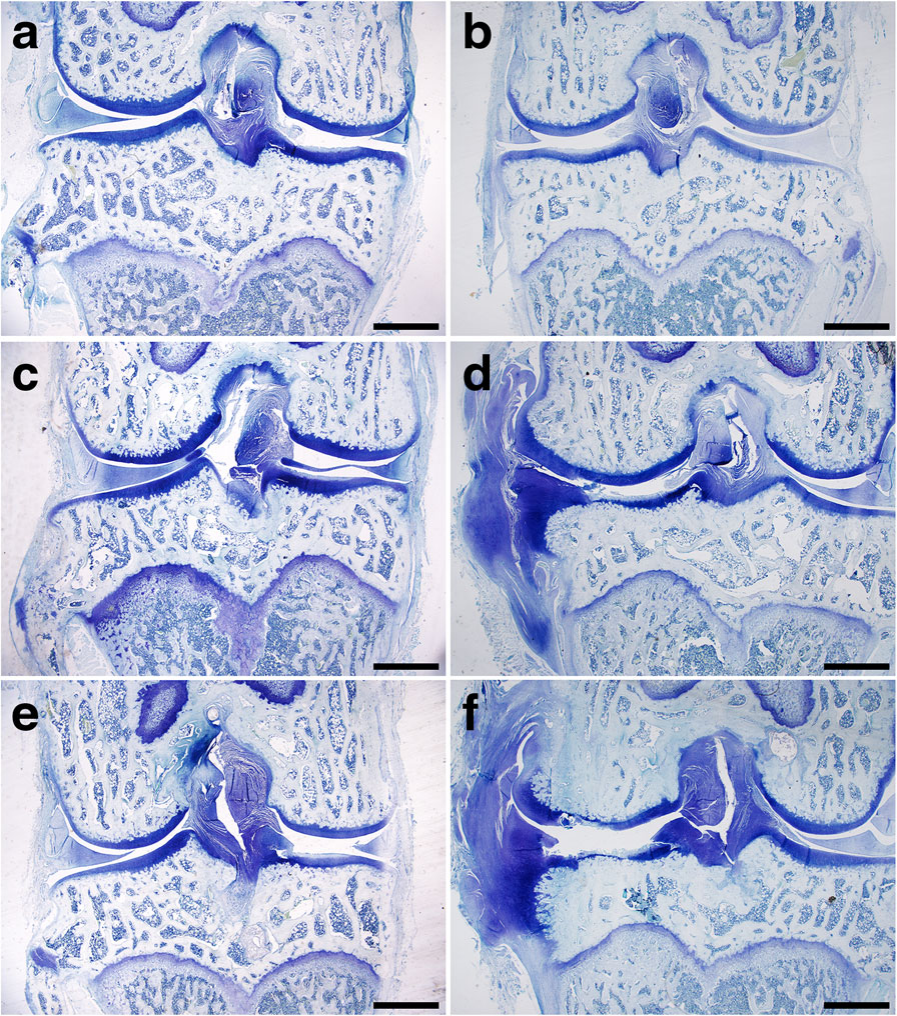
OARSI Microscopic scoring among groups. Notes: Toluidine *blue stain* of medial tibial plateau of samples, 40×. Sham group: (**a**) for left knee and (**b**) for Right knee; MNX + V group: (**c**) for left knee and (**d**) for right knee; MNX + SR group: (**e**) for left knee and (**f**) for right knee. Bars represent length of 1 mm

Histological data for cartilage in the medial tibial plateau also indicated that cartilage in the load bearing area of the MNX + V group was severely injured (Fig. 3), with loss of cartilage or clefts extending to calcified cartilage, as evidenced by decreased toluidine blue staining in middle and deeper zones, the formation of cellular clusters, and large chondral hyperplasia on the medial joint edge with osteophytes underlying it. Total scores for the MNX + V or MNX + SR groups were significantly higher than for shams but there were no significant differences between MNX + SR and MNX + V groups (*P* = 0.297). The results showed significantly reduction of cartilage thickness in MNX groups versus sham group (Fig. 2f). Data indicate (Table 1) that SR increased osteophyte sizes compared to those in the MNX + V group (*P* = 0.014). Two MNX + SR samples had collapsed osteophytes, and no osteophytes collapsed in the MNX + V group, so there was no significant difference between the MNX + SR and MNX + V groups (*P* = 0.455).

**Table 1:** Osteophyte measurements and collapsed osteophytes

Group	D1 (*n* = 6, μm)	D2 (*n* = 6, μm)	Osteophyte size (D1-D2, *n* = 6, μm)	Collapsed osteophytes		
				in macroscopic samples (*n* = 6)	in histologic samples (*n* = 6)	in all samples (*n* = 12)
Sham	263.62 [231.18, 296.06]	280.26 [247.42, 313.10]	-16.64 [-39.56, 6.28]	0	0	0
MNX + V	370.19 [330.19, 410.19]^*^	263.33 [234.28, 292.39]	106.86 [46.93, 166.79]^*^	1	0	1
MNX + SR	445.97 [415.36, 476.58]^*†^	247.79 [212.40, 283.17]	198.18 [147.48, 248.88]^*†^	5	2	7^*†^

^*^ *vs*. Sham group, *P* < 0.05

^†^ *vs*. MNX + V group, *P* < 0.05

Seven samples had collapsed osteophytes from all twelve animals in the MNX + SR group but only one sample was collapsed in MNX + V group. There was a statistical difference between SR-treated animals and MNX-operated saline-treated animals (*P* = 0.027), and the risk ratio for osteophyte collapse in the MNX + SR group was 7.00 [1.01, 48.54].

### Immunohistochemical analysis

**Fig. 4:**
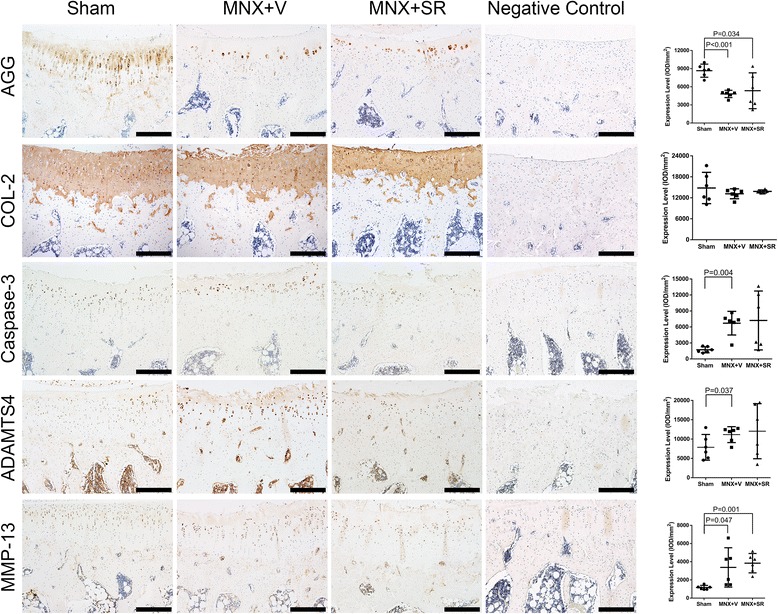
Immunohistochemical staining of load bearing zone of tibial plateau of groups. Notes: 100×, bars represent a length of 400 μm

The expressions of AGG, Col-II, ADAMTS-4, MMP-13, and caspase-3 protein in the tibial plateau were measured and Fig. 4 shows that AGG expression in MNX + V and MNX + SR groups was significantly lower than those in sham-operated animals but MMP-13 expression was greater than that in the sham animals. ADAMTS-4 and caspase-3 were significantly higher in the MNX + V group and there were no significant differences in expression of collagen type II among the three groups (*P* = 0.553).

**Fig. 5:**
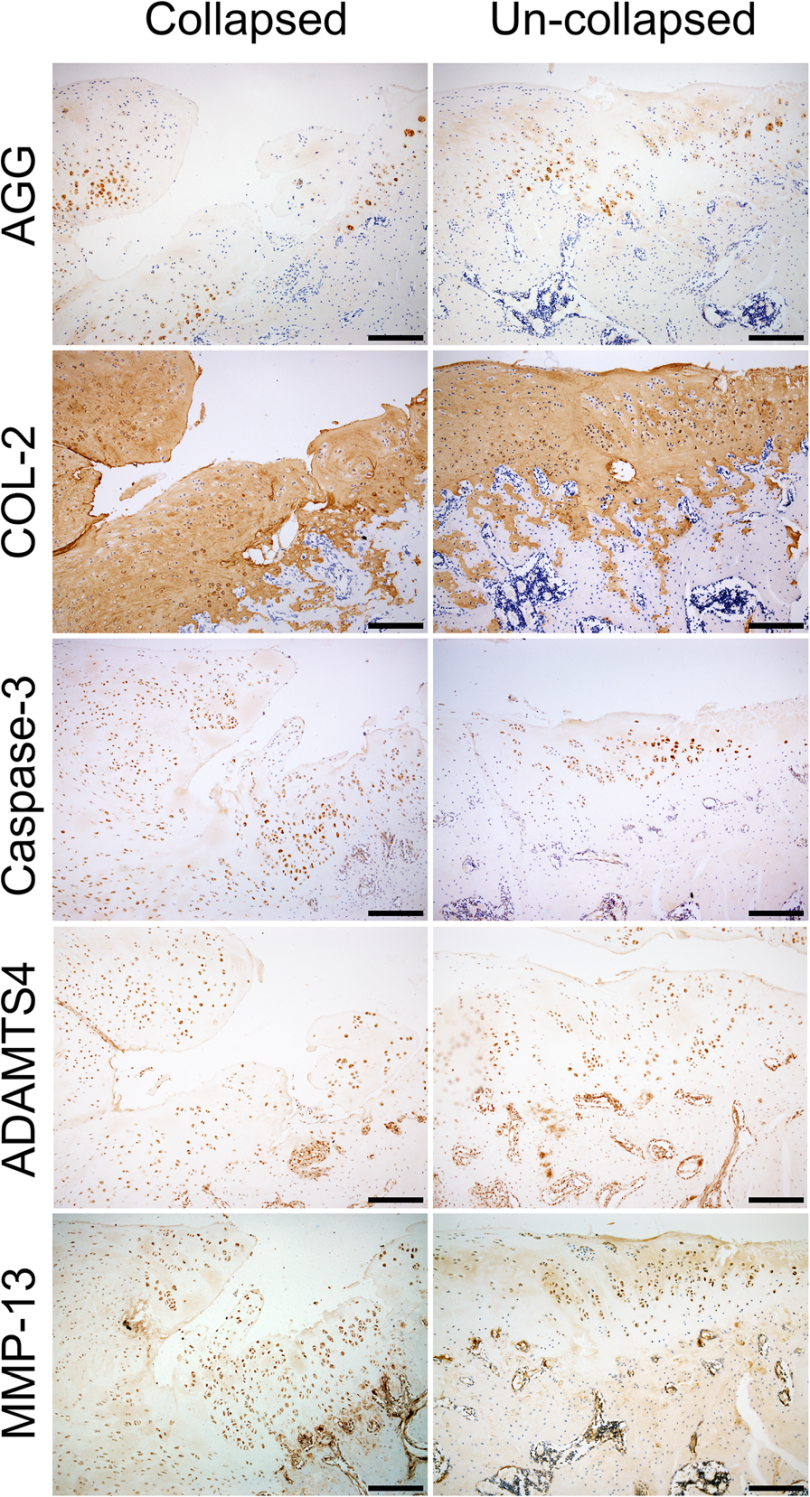
Immunohistochemical staining of cartilage at the transitional place between osteophyte and plateau cartilage. Notes: 100×, *bars* represent a length of 400 μm

Immunohistochemistry indicated that extracellular matrix components AGG and Col-II were absent in chondrocytes in the center of collapsed osteophytes, whereas positively stained chondrocytes of apoptosis related protein caspase-3 and degenerative proteins ADAMTS4 and MMP-13 were observed (Fig. 5). For un-collapsed samples, an absence of positively stained chondrocytes of AGG and Col-II were also observed in the superficial layer of the transitional area between the tibial plateau and osteophyte, whereas caspase-3, ADAMTS4 and MMP-13 positively stained chondrocytes were observed. Alignments of chondrocytes were diffused radially from the base to the surface of cartilage at the transitional place between osteophyte and plateau cartilage.

### Micro-architecture parameters of subchondral bone

**Fig. 6:**
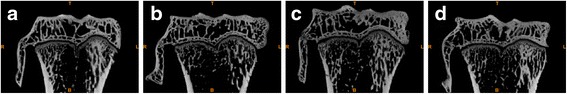
Reconstruction of coronal section of right tibial plateau from each group. Notes: Sham group: (**a**); MNX + V group: (**b**); MNX + SR group with collapsed osteophyte: (**c**); MNX + SR group with un-collapsed osteophyte: (**d**)

**Fig. 7:**
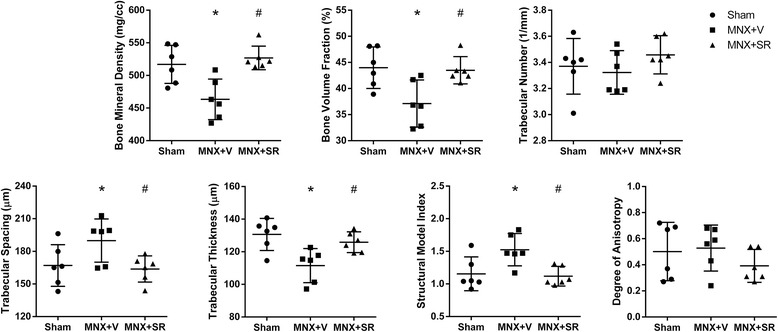
Histomorphometric parameters of subchondral bone by micro-CT analysis. Notes: * *vs*. Sham group, *P* < 0.05; # *vs*. MNX + V group, *P* < 0.05

Figures 6 and 7 show significantly less BMD in the MNX + V group compared to shams (*p* = 0.002). However, BMD was significantly greater in the MNX + SR group (*p* = 0.001). Other subchondral bone micro-architecture parameters (BV/TV, TB.Sp, Tb.Th, and SMI) indicated subchondral bone loss in the MNX + V group compare to shams. However, Tb.N (*P* = 0.404) and DA (*P* = 0.368) were not different among groups. Condensed bone tissue was noted in the sample of collapsed osteophytes.

## Discussion

In this study, although subchondral bone was significantly enhanced by SR (625 mg/kg/day), it did not protect cartilage at this dose from degeneration in meniscectomized guinea pigs. Besides, SR stimulated osteophytes overgrowth with cleft formation and/or osteophyte collapse in meniscectomized guinea pigs. The dose used in this study, considering strontium bioavailability would be reduced by ~60–70% if taken with calcium or food, is similar to that used by Pelletier et al. [15]. They demonstrated therapeutic effects of 75 mg/kg/day in ACLT dogs at least 2 h before feeding. Yu et al. [16] reported that 1,800 mg/kg/day showed therapeutic effects in MNX rats. Bruel et al. [22] showed that mean serum Sr of 8990 μg/L results from 8 weeks of 900 mg/kg/day for rats, thus serum Sr in Yu’s study should be no less than this value. On the contrary, Meunier et al. [23] reported the mean serum Sr is 117.9 μg/L in patients who regularly receive SR at 2 g/day. Because a potential risk of venous thromboembolism (VTE) may occur in SR-treated patients [24], high SR dose may encounter obstacles when adapting the drug to human OA treatment. In this view, a lower dose as in this study and Pelletier et al.’s tends to be more appropriate.

Although several in vivo studies are described in the literature [15, 16], none have focused on SR in a guinea pig model which has been described by Bendele in detail [19]. This model is similar to human OA, and features cartilage degeneration, osteophytes formation, and fibroblast proliferation in the synovium. Surgically induced OA models, such as anterior cruciate ligament transectomized (ACLT) [25] and MNX animals, generate cartilaginous lesions rapidly but different surgical approaches cause various rates of cartilage degeneration. Generally, cartilage in the MNX model degenerates more rapidly than in the ACLT model due to more joint instability [19]. Thus, the MNX model might narrow the therapeutic window for drug intervention. And they might be obscured if there exist any potential therapeutic effects of SR.

Instead, SR stimulated osteophyte growth and subsequent collapse. The method of osteophyte measurement is adapted from the method for rats reported by Gerwin et al. which only measured cartilaginous tissue [26]. Osteophytes often grow upward and outward on tibial plateau edge and guinea pigs’ osteophytes contain more bony tissue, so data from this method should be more representative with respect to the extent of osteophytes growth direction. Our data show that osteophytes were larger in SR-treated animals, which indicates that osteophytes may be stimulated by SR. This finding is supported by work published by Panahifar et al. [6] who used SR as a tracer and mapped strontium with electron probe microanalysis (EPMA). They reported strontium incorporated toward the osteophytic margins in meniscectomized rats. Panahifar et al. further reported that strontium was heavily incorporated in mineralizing osteophytes at 4, 8, and 12 weeks post-surgery, whereas it was incorporated in subchondral bone only between weeks 8-12 [18]. However, an earlier study in which radioactive strontium isotope ^85^Sr was used as a tracer for monitoring patella bone turnover in the medial or lateral femorotibial articulation in severe symptomatic OA patients [27], suggesting that osteophytes were not associated with high uptake of ^85^Sr. These contradictory data may be explained by different phases of OA progression aimed in these studies. Panahifar et al. suggested that in early phases of OA progression, rather than the knee plateau, osteophytes have greater affinity for strontium. Furthermore, SR can strongly stimulate human cartilage matrix formation in vitro by a direct ionic effect [28]. From strontium accumulation, chondrocytes in osteophytes could be specifically stimulated and the chondral aspect of the osteophyte grows, forming larger osteophytes in early OA. Chondrocytes in the knee plateau were merely stimulated, thus cartilage degeneration may be unaffected.

Collapsed osteophytes observed here were related to condensed subchondral bone beneath the osteophyte, not only related to osteophyte overgrowth stimulated by SR. Figures 3f and 6c show that canals almost disappeared in bony osteophytes and the subchondral bone plate. Condensed subchondral bone could transmit increased loads to overlying cartilage, although its reason is unclear, leading to further cartilage degeneration [29]. In contrast, because the articular cartilage is avascular, the permeability of calcified cartilage and subchondral bone plates allows crossover communication, providing connecting channels between subchondral bone and cartilage [30]. Canal reduction in subchondral bone or osteophytes would affect nutrition and oxygen and induce chondrocyte apoptosis, which could contribute to osteophyte collapse.

It was noted that chondrocytes alignments diffused radially from the cartilage-subchondral bone interface to the cartilage surface at the transitional area between plateau and osteophyte. This suggests that this region is under abnormal mechanical stress, which may be due to stretching by osteophyte and plateau cartilage. This transverse force may be derived from the bearing load and transduced by excessively grown osteophytes. Then, this region would have exhibits catabolic changes. AGG and Col-II, two major components of the cartilage matrix [31, 32], were absent in chondrocytes located in the center of collapsed osteophytes, and this may suspend the repair process. Also, ADAMTS4 and MMP-13, enzymes responsible for degrading AGG and Col-II [33, 34], were expressed by chondrocytes, which will accelerate cartilage degeneration locally. These findings were supported by Su et al. [35] who reported that an in vitro model mimics mechanical stress and undergoes similar changes in chondrocytes. Abnormal mechanical stress can also induce chondrocyte apoptosis, which was shown by increased expression of caspase-3 which is associated with mechanical stress according to Kong et al. [36]. Thus, these changes contribute to the formation of a cleft in the region between the osteophyte and the knee plateau.

The limitation of the current study include the small number of animals and the single time point. To avoid diffused staining in immunohistochemistry procedure caused by 100% ethanol fixation, we had to randomly divide each group further into two subgroup. Consequently, the number of animals available for microCT and immunohistochemistry analysis were reduced. Another consequence is randomization could not completely eliminate sampling error between subgroups, which result in potential weaken the power of results. The study conducted on a surgical induced OA model, the progress of cartilage lesion is rapid. Single time point observation may be insufficient to discover potentially existed therapeutic effect of SR. And the last, the secondary outcomes, such as osteophyte overgrowth and osteophyte collapse, should be tested and verified by further research.

## Conclusions

In conclusion, SR (625 mg/kg/day) did not protect cartilage from degeneration in an MNX guinea pig model but subchondral bone was significantly enhanced by SR treatment. In this early phase OA model, SR causes osteophyte overgrowth and this may be due to its ability to incorporate into osteophytes and promote hyperplasia. This adverse effect should be monitored in future SR studies of OA.

## Abbreviations

ADAMTS-4: a disintegrin and metalloproteinase with thrombospondin motifs-4
AGG: aggrecan
BMD: bone mineral density
BV/TV: bone volume fraction
Coll-II: collagen type II
IOD: integrated optical density
MMP-13: matrix metalloproteinase-13
MNX: medial meniscectomized
OA: osteoarthritis
SMI: structural model index
Tb.N: trabecular number
Tb.Sp: trabecular spacing
Tb.Th: trabecular thickness
